# Hydrogen Production During Ethylene Glycol Photoreactions Over Ag-Pd/TiO_2_ at Different Partial Pressures of Oxygen

**DOI:** 10.3389/fchem.2019.00780

**Published:** 2019-11-22

**Authors:** Ahmed Khaja Wahab, Mohammad Amtiaz Nadeem, Hicham Idriss

**Affiliations:** Hydrogen Platform, Catalysis Department, SABIC Corporate Research and Development (CRD), King Abdullah University for Science and Technology (KAUST), Thuwal, Saudi Arabia

**Keywords:** photo-catalytic hydrogen production, photo-oxidation of ethylene glycol, Ag-Pd based catalyst, formaldehyde intermediate, carbon-carbon bond dissociation

## Abstract

The reaction of ethylene glycol has been studied over Ag–Pd/TiO_2_ (anatase) under photo-irradiation while monitoring the reaction products (in the gas and liquid phases) as a function of time and at different partial pressures of molecular oxygen. The catalyst contained metal particles with a mean size of about 1 nm, most likely in the form of alloy (TEM, STEM, and XPS). The complex reaction network involves hydrogen abstraction, C-C bond dissociation, de-carbonylation and water gas shift ultimately yielding hydrogen and CO_2_. The two main competing reactions were found to be, photo reforming and photo-oxidation. Based on our previous study, Ag presence improves the reaction rate for hydrogen production, most likely via decreasing the adsorption energy of CO when compared to pure Pd. At high ethylene glycol concentrations, the rate of hydrogen produced decreased by a factor of two while changing O_2_ partial pressure from 0.001 to 0.2 atm. The rate was however very sensitive to oxygen partial pressures at low ethylene glycol concentrations, decreasing by about 50 times with increasing oxygen pressures to 1 atm. The order of reaction with respect to O_2_ changed from near zero at high oxygen partial pressure to ½ at low partial pressure (in 0.008–0.2 atm. range). Liquid phase analysis indicated that the main reaction product was formaldehyde, where its concentration was found to be higher than that of H_2_ and CO_2_. The mass balance approached near unity only upon the incorporation of formaldehyde and after a prolonged reaction time. This suggests that the photo-reforming reaction was not complete even at prolonged time, most likely due to kinetic limitations.

## Introduction

Hydrogen can be produced from clean and renewable energy sources, its oxidation releases 122 kJ/g of energy which is about 2.7 times higher than a typical hydrocarbon fuel combustion (Balat, 2008). It can be produced from ethanol and glycols; both are derived from fermentation or hydrolyses of cellulose, ensuring its renewable cycle. While aqueous phase reforming of glycols at high temperatures is feasible, it may not be sustainable because fossil fuel needs to be burned to provide the needed reaction energy. Photocatalytic hydrogen production using sun light might be more attractive because in this case the cycle would be sustainable. Also, ethylene glycol and glycerol usually exist as aqueous solution in petrochemical industrial waste streams and their separation is difficult due to their hydrophilic nature, therefore these kind of wastes can be utilized for the photocatalytic hydrogen production (Kim and Hoffmann, 2008).

When TiO_2_ is irradiated by incident light with energy above its band gap, electrons are excited from the valence band (VB) into the conduction band (CB) producing electron-hole pairs. Recombination of these pairs takes place in competition with the charge transfer process during catalysis; with the former being orders of magnitudes faster than the latter (Hoffmann et al., 1995). This fast recombination rate decreases the photocatalytic activity and results in low quantum efficiency (Philip Colombo et al., 1995; Colombo and Bowman, 1996). Depositing on TiO_2_ noble metals such as Pt, Pd, and Au increases the reaction rate, most likely, via trapping of CB electrons, providing active sites for proton reduction (Al-Azri et al., 2015). Production of hydrogen should be accompanied by oxygen evolution for the complete water splitting reaction. In the case of TiO_2_ oxygen evolution reaction is hindered due to the formation of peroxides among other reasons (Daskalaki et al., 2011; Alghamdi and Idriss, 2018). To overcome these problems sacrificial electron donors such as alcohols and glycols are used to inject electrons into the VB of TiO_2_.

The photocatalytic reaction of ethylene glycol in aqueous media over metal-doped TiO_2_ would involve some of the following steps. (1) Electron injections into the VB of TiO_2_ (commonly referred to as hole transfer), this step allows more excited electrons to reduce hydrogen ions (Murdoch et al., 2011). (2) OH^**·**^ radicals are formed upon injection of one electron into the VB from a surface hydroxyl group (Ollis et al., 1991). (3) Hydrogen atoms abstraction from ethylene glycol by the photo generated OH^**·**^ radicals (Legrini et al., 1993). OH^**·**^ radicals have a redox potential of +2.81 V vs. SHE (standard hydrogen electrode) and act as a main oxidizing agent in the degradation of organics. The formation of OH^**·**^ radicals from H_2_O proceeds with the release of one H^+^ ion that can accept electrons from the CB to form hydrogen. The following equations show the OH^**·**^ radical formation and H^+^ ion reduction (written as surface hydroxyls).

(1) $$2{\text{H}}_{2}\text{O}(\text{a})\;+\;2\text{h }+\;2O(\text{s})\to \;2{\text{OH}}^{\cdot }\;+\;2O\text{H}(\text{s})$$

(2) $$2O\text{H}(\text{s})\;+\;2\text{e}\to {\text{H}}_{2}\;+\;2O(\text{s})$$

Where (a) and (s) stand for adsorbed state and surface site, respectively. e and h stand for excited electron and hole, respectively.

The oxidation of ethylene glycol can also occur via direct electron injection into the VB of TiO_2_ (Al-Azri et al., 2015). Whether the oxidation of ethylene glycol takes place directly by VB holes or by OH^**·**^ radicals or both, the overall reaction in anaerobic (photo-reforming) condition is the complete reforming of ethylene glycol to CO_2_ and H_2·_ The complete photo-reforming equation of ethylene glycol is expressed by the following equation.

(3) $${\text{C}}_{2}{\text{H}}_{6}{\text{O}}_{2}\;+\;2{\text{H}}_{2}\text{O}\to 2{\text{CO}}_{2}\;+\;5{\text{H}}_{2}\;\Delta \text{H}{}^{\circ}=\;+90{\text{kJmol}}^{-1}$$

Photo generated electrons in TiO_2_ are trapped by Ti^4+^ sites to form Ti^3+^ (Komaguchi et al., 2009). In the presence of O_2_ the Ti^3+^ is readily oxidized by oxygen to form anionic superoxide radical O_2_^·−^ (Komaguchi et al., 2009)_·_ The redox potential of O_2_^·−^ is +0.89 V vs. SHE. Superoxide anions can be protonated to HO_2_· in acidic solution and have pK_a_ value of 4.8 (Colmenares and Luque, 2014), they can also react with adsorbed organic species or free radicals in the solution (Braun and Oliveros, 1997; Carp et al., 2004; Mills et al., 2006). The complete oxidation of ethylene glycol proceeds by the following equation.

(4) $${\text{C}}_{2}{\text{H}}_{6}{\text{O}}_{2}\;+\;5/2{\text{O}}_{2}\to 2{\text{CO}}_{2}\;+\;3{\text{H}}_{2}\text{O}\Delta {\text{H}}^{{}^{\circ}}=-1,120{\text{kJmol}}^{-1}$$

Huber et.al summarized the aqueous phase reforming reactions of ethylene glycol, where C-C, C-H and O-H bonds cleavage occurs producing adsorbed intermediates (Huber et al., 2006). Another competing reaction is the photo-oxidation of glycols to CO_2_ and water in the presence of O_2_ which decreases the total yield of H_2_. The photo-oxidation and photo-reforming of glycerol, a compound that has many structural and redox potential similarities to ethylene glycol, over Pt-TiO_2_ was also studied by Panagiotopoulou et.al (Panagiotopoulou et al., 2013). Their results showed that in photo-oxidation the main products were CO_2_ and water while in the case of photo-reforming these switched to H_2_ and CO_2_. The photo-oxidation of several organics including phenols and alcohols over TiO_2_ have been studied in good details by many authors (Matthews, 1988; Ollis, 2005). The reaction rates are often fitted by Langmuir-Hinshelwood kinetic expressions. It was also found that the Langmuir adsorption constants in photoreaction conditions are different from those found in the dark. This is most likely due to the shift in the adsorption constants of organic compounds in the presence of additional energy; the adsorption constant of molecular oxygen was found to be independent of light intensity, however, most likely due to its weak adsorption over TiO_2_ (Mills et al., 2006).

In this work, we have studied the photo-reforming of ethylene glycol (EG) at various partial pressures of O_2_ (*P*_O2_) over 0.1 wt.%Ag−0.3 wt.%Pd-TiO_2_ (anatase with particles size of about 10 nm). This catalyst was found in separate studies to be among the most active for hydrogen production (Wahab et al., 2016; Nadeem et al., 2017). The different products (in the gas and liquid phases) during the photoreaction of ethylene glycol were followed in order to understand the competition between photo reforming and photo-oxidation of ethylene glycol using Langmuir-Hinshelwood type kinetics.

## Experimental

### Catalysts Preparation

TiO_2_ (anatase) (Hombikat by Sachtleben Chemie GmbH) was used as a support, AgNO_3_ (Sigma Aldrich 100%) and Pd(CH_3_COO)_2_ (Sigma Aldrich 99.9%) were used as precursors for Ag and Pd, respectively. The desired amount of aqueous stock solution of AgNO_3_ and Pd(CH_3_COO)_2_ were deposited by the impregnation method on the support. The mixture of TiO_2_ and metal salts were stirred and heated at 180–200°C for 12–24 h in a round bottom flask equipped with a condenser. The mixture was then poured into an empty beaker and heated over a heating plate until all water has evaporated while stirring. The resulting solid was then scratched out of the beaker using a glass rod and dried in an oven at 100–110°C for 12 h followed by calcination at 350°C for 5 h.

### Catalysts Characterization

TEM analyses were performed with Titan ST transmission electron microscope equipped with a field emission electron source. It was operated at 300 kV. Samples were deposited from alcohol suspensions on to holey-carbon Cu grids. The microscope was operated either in HRTEM (phase contrast) or HAADF-STEM mode (Z-contrast). The point-to-point resolution was 0.12 nm and the information limit was 0.10 nm. HRTEM beam focus was 100 nm while STEM was 1.0 nm. Energy–dispersive X–ray spectroscopy (EDX) were carried in STEM mode of operation. UV-Vis absorbance spectra were collected over the wavelength range of 250–900 nm with a Thermo Fisher Scientific UV-Vis spectrophotometer equipped with a praying mantis diffuse reflectance accessory. The measurements were conducted for the fresh catalysts as well as for one reduced under hydrogen at one atmosphere at 250°C for 5 h. XPS measurements were conducted using a Thermoscientific ESCALAB 250 Xi, equipped with a mono-chromated Al K_α_ X-ray source. The base pressure of the chamber was typically in the low 10^−10^ to high 10^−11^ mbar range. Charge neutralization was used for all samples (compensating shifts of ~1 eV). Spectra were calibrated with respect to C1s at 285.0 eV. The Ag3d, Pd3d, Ti2p, C1s, and valence band binding energy regions were scanned. Typical acquisition conditions were as follows: pass energy = 30 eV and scan rate = 0.1 eV per 200 ms. Self-supported disks of approximately 0.5 cm diameter were loaded into the chamber for analysis. Ar ion bombardment was performed with an EX06 ion gun at 1 kV beam energy and 10 mA emission current; The sputtered area of 900 × 900 μm^2^. A typical spatial area analyzed was 0.65 × 0.65 mm^2^. BET surface area of TiO_2_ (anatase) calcined at 350°C for 5 h is equal to 130.6 m^2^/g with a pore volume of 0.36 cm^3^/g and an average pore size of 11 nm (ASAP 2050 XP, Micrometrics). XRD was conducted using a Panalytical Empyrean series 2-XRD at the following condition: A 2θ interval between 5 and 90θ was used with a step size of 0.013 and a step time of 0.1 s. The X-ray, Ni-filtered Cu Kα radiation source (K_α_ = 1.5418 Å), operated at 45 mA and 40 kV.

### Photoreactions Setup

Photoreactions were performed in 140 mL glass reactor. The reactor was flat from all sides to avoid the convergence or divergence of light. The catalyst was dispersed in 20 mL water and bubbled with O_2_ for the required *P*_O2_ in the gas phase. Ethylene glycol was added into the reactor to get the required concentration with keeping the total volume of the aqueous mixture 20 mL. The final mixture was subjected to constant stirring initially under dark condition for 30 min for better catalyst and ethylene glycol dispersion. The reactor was then exposed to the UV light by a 100 Watt ultraviolet lamp (H-144GC-100, Sylvania par 38) with a flux of ca. 5 mW/cm^2^ at a distance of 10 cm with the cut off filter (360 nm). Gas phase product analyses were performed by Agilent 7890A Gas Chromatography (GC) equipped with Thermal Conductivity Detector (TCD) on a Haysep Q packed column at 45°C and N_2_ was used as a carrier gas. Oxygen detection was conducted using a molecular sieve 5A column with TCD detector and He carrier gas at 80°C. For the analysis of liquid intermediates, GC-MS (Thermo Fisher Tracer 1300) equipped with a capillary column CP-WAX CB 57 (50 m long) was used. Helium was used as a carrier gas and the temperature was ramped from 100 to 250°C at the rate of 10°C per minute. Liquid samples were injected manually using a syringe with sample injection of 0.2 micro liter at a split ratio of 20.

## Results

**Figure 1:**
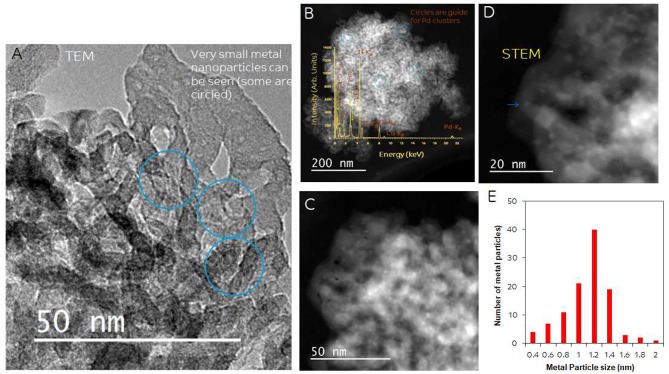
**(A)** TEM of 0.1 wt.% Ag-TiO_2_, 0.1 wt.% Ag-0.3 wt.% Pd-TiO_2;_ the blue circles show some of the dark spots due to Pd and Ag. **(B)** STEM-HAADF (High-Angle Annular Dark-Field) image, inset: EDX of metal particles; **(C,D)** Higher magnification STEM showing Pd/Ag particle dispersion on top of TiO_2_. **(E)** Particle size distribution.

**Figure 2:**
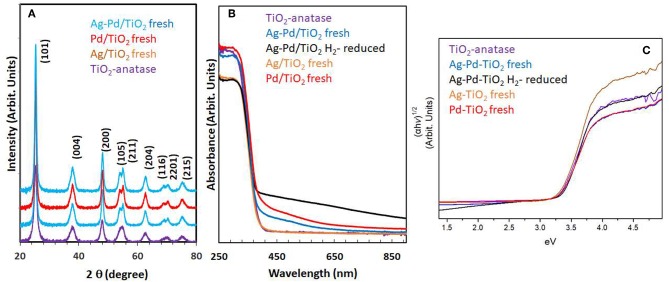
**(A)** XRD of the TiO_2_ (anatase), Ag/TiO_2_, Pd/TiO_2_, and Ag-Pd/TiO_2_; **(B)** UV-Vis absorbance of TiO_2_, Ag-TiO_2_, Pd/TiO_2_, Ag-Pd-TiO_2_ fresh, and H_2_-reduced; **(C)** The corresponding Tauc plots of the data of **(B)**.

Particle size and distribution were analyzed by TEM over this TiO_2_ (anatase); the XRD of TiO_2_ is presented in Figure 2A. Catalysts consisted of well-dispersed metal nanoparticles of mean size about 1 nm over the TiO_2_ support with size of about 10–15 nm. Figure 1A shows A TEM image of the catalyst. The small particle size and the usual low contrast between TiO_2_ and Pd (or Ag) makes the identification difficult (Al-Azri et al., 2019). Yet, clear dark spots can be seen. These are better analyzed by STEM-HAADF (High-Angle Annular Dark-Field). Figure 1B presents a low magnification STEM. The even distribution of metals on top of TiO_2_ is clear (brighter spots) further confirmed with EDX (inset of Figure 2A) where the presence of Pd and Ag is identified by their L_α_ lines at 2.93 and 2.98 keV. Note that the Pd signal is larger than that of Ag (0.1 wt.% Ag−0.3 wt.% Pd). The metal nanoparticles are recognized as bright dots, according to their higher atomic weight with respect to TiO_2_. Figures 1C,D shows two magnifications of the STEM images. Particles are well-seen, in most cases it was not possible to see differences between them indicating that most likely an alloy of Pd and Ag is made. Particle size distribution is given in Figure 1E. Particles as small as 0.5 nm are seen and the mean size is about 1 nm. UV-Vis spectra of blank TiO_2_ (anatase) and the mono- and bi-metal deposited TiO_2_ (anatase) catalysts are shown in Figure 2B. The wide band gap of TiO_2_ (anatase) at about 3.2 eV and above does not change due to the presence of the different metals (Ag, Pd) or their alloy (Pd-Ag). There is however some absorption in the visible light. This is not due to the plasmonic of Ag (probably because of its very small amount 0.1 wt.%) but mostly due to Pd cations of Pd-O due to d-d electronic transitions (Nadeem et al., 2017). It is worth noting that the visible response is more accentuated for the prior-hydrogen reduction bimetal catalyst; this is due to bulk defects and may not be related to an increase in the photocatalytic activity as they can act as charge carrier trap centers. Figure 2C shows the Tauc plots of the same series of catalysts in Figure 2B. TiO_2_ anatase is an indirect-band gap semiconductor and is therefore modeled using the Tauc plot as follow:

$$\text{α}h\nu =\text{A}{\left(h\nu -{\text{E}}_{g}\right)}^{\text{n}}$$

Where a is the absorption coefficient, A a constant, n the light frequency, E_g_ the band gap to be extracted and n denotes the nature of the electronic transition. *n* = ½ for a direct allowed transition, = 3/2 for direct forbidden transition, = 2 for an indirect allowed transition and = 3 for indirect forbidden transition within a semiconductor material. In the case of TiO_2_ (anatase) *n* = 2. Taking the square root of both sides yields

$${\left(\text{α}h\text{ν}\right)}^{1/2}=A^{\prime }\left(h\text{ν}-\;E_{g}\right)$$

Where A′ is a constant. Therefore, plotting (α*h*ν)^1/2^ as a function of *h*ν gives a straight line with E_g_ as the intercept. Marginal differences in the band gap of TiO_2_ are seen due to the presence of the metals on top of the support. Ag nanoparticles have plasmonic resonance response in the UV and visible region, depending on size, morphology and dielectric constant of the medium (Linic et al., 2015). We have studied these type of catalysts in good details by XPS and UV-Vis absorbance (Nadeem et al., 2017), previously. In this work, while the amount of Ag and Pd is too small, it can be seen that the hydrogen-reduced Ag-Pd/TiO_2_ bimetallic catalyst has an increased absorbance in the visible region most likely due to the presence of both metals; formed upon reduction (of PdO and Ag_2_O to Pd and Ag) with hydrogen.

**Figure 3:**
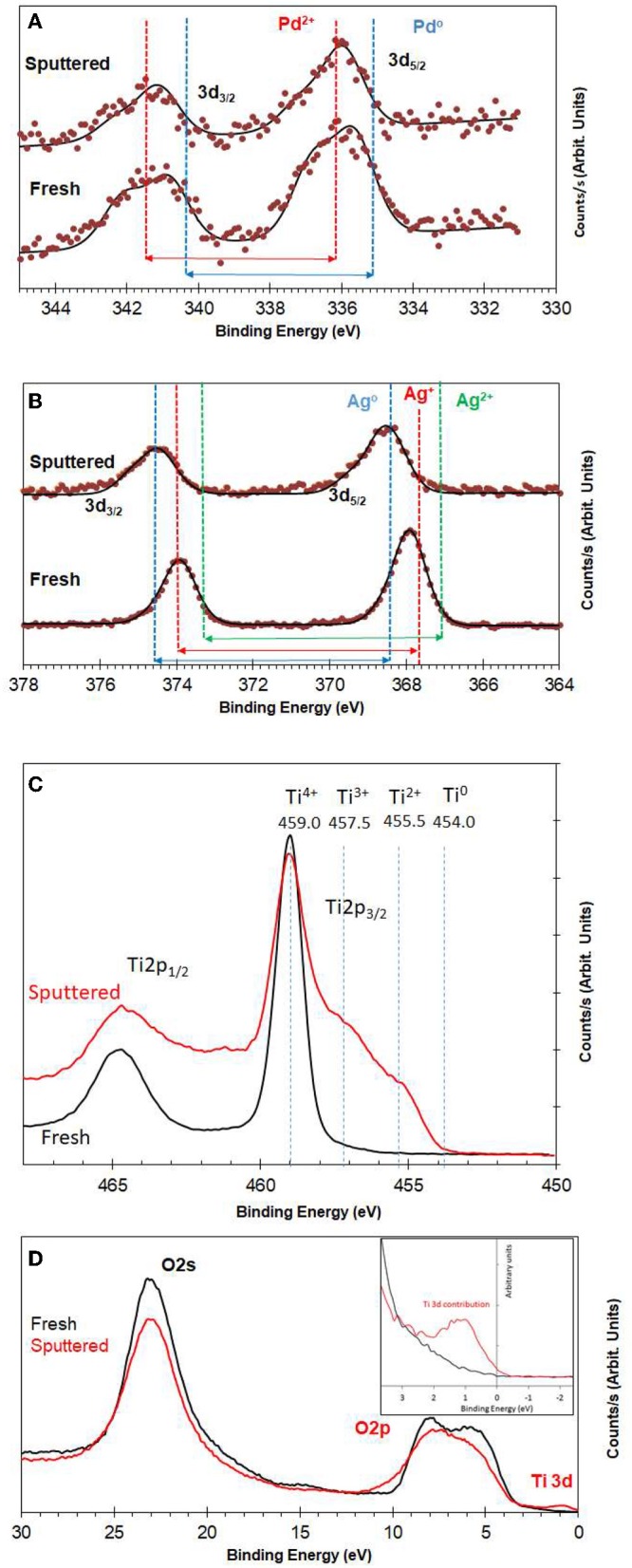
**(A)** XPS Pd3d for fresh and Ar^+^-bombarded 0.1 wt.% Ag−0.3 wt.% Pd/TiO_2_ (anatase) catalyst; **(B)** XPS Ag3d for fresh and Ar^+^-bombarded 0.1wt.% Ag−0.3 wt.% Pd/TiO_2_ (anatase) catalyst; **(C)** XPS Ti2p for fresh and Ar^+^-bombarded of 0.1 wt.% Ag−0.3 wt.% Pd/TiO_2_ (anatase) catalyst. **(D)** Valence band region for fresh and Ar^+^-bombarded 0.1 wt.% Ag−0.3 wt.% Pd/TiO_2_ (anatase) catalyst. Quantitative analysis indicates that Pd, and Ag are in 0.1 and 0.25 at %. The atomic and weight ratios of the sum of the metals (Pd + Ag) to Ti cations are 0.01 and 0.023, respectively. These calculations are based on the integrated peak areas corrected by the ionization cross section of the elements (Ti2p, Pd3d, and Ag3d) which are 1.8, 4.6, and 5.2 with respect to F1s.

Figure 3A shows XPS spectra of Pd3d of 0.1 wt.% Ag−0.3 wt.%Pd before and after Ar ions sputtering. We used Ar ions to mimic the reduced states of the catalysts. Ar ions results in the preferential removal of oxygen ions as it relies mostly on mass difference (Ag and Pd are one atomic mass unit apart) (Yamamura, 1982). It is often used to study the extent of reduction of metal oxide (Idriss and Barteau, 1994; Chong et al., 2001). More details on the changes in the core levels of Ag and Pd can be found in Nadeem et al. (2017) and here we give a brief description. Pd was present in two oxidation states (Pd^2+^ of PdO and Pd^0^); binding energies at 336.0 and 335.0 eV, respectively for Pd 3d_5/2_ (Gallo et al., 2012; Bashir and Idriss, 2017). The spin orbit splitting between 3d_3/2_ and 3d_5/2_ for PdO and Pd is 5.5 and 5.3, respectively. The signal from Pd metal at 335 eV increased due to reduction after 5 min of Ar ions sputtering. Figure 3B presents XPS spectra of Ag3d before and after Ar ions sputtering. In the as prepared catalyst, Ag was mainly present in the form of Ag_2_O and AgO with Ag 3d_5/2_ binding energy positions at 367.4 and 367.9 eV with spin orbital splitting of 5.9 and 6.0 eV, respectively. Quantitative analysis of the Pd3d, Ag3d, and Ti2p peak areas indicates that the surface and near surface is richer in in Ag than in Pd. This might be because of the smaller particle size of Ag when compared to Pd. The Ar ions sputtered samples show increase in signals of Ag metal (the unusual positive binding energy shift with reduction is typical of Ag core level spectroscopy Gaarenstroom and Winograd, 1977; Weaver and Hoflund, 1994) due to reduction of the oxide form to the metallic state as in the case of Pd. Figure 3C presents the reduction of TiO_2_ upon argon ion sputtering where the Ti2p lines have considerably broadened due to the formation of multiple oxidation states as a result of oxygen ions removal. This results have seen and studied before (Idriss and Barteau, 1994; Idriss et al., 1996; Fleming et al., 2007, among others). Figure 3D presents the valence band spectra, because of the region is very sensitive to the any changes in the chemical bonding we have opted to align it with the O2s line at 23.0 eV. The valence band spectra of the fresh and sputtered catalysts were compared to see any changes due to the presence of Ti3d states associated with oxygen defects. The reduced sample showed a small peak (at about 1 eV below the Fermi level) due to the presence of electrons in the Ti3d band (Bashir et al., 2015; Bashir and Idriss, 2017).

Next, the photo reforming of ethylene glycol on this catalyst is presented with a focus on the effects of reactant concentrations and of oxygen partial pressures.

### Photoreactions at High Concentrations of Ethylene Glycol (5 vol.%)

**Figure 4:**
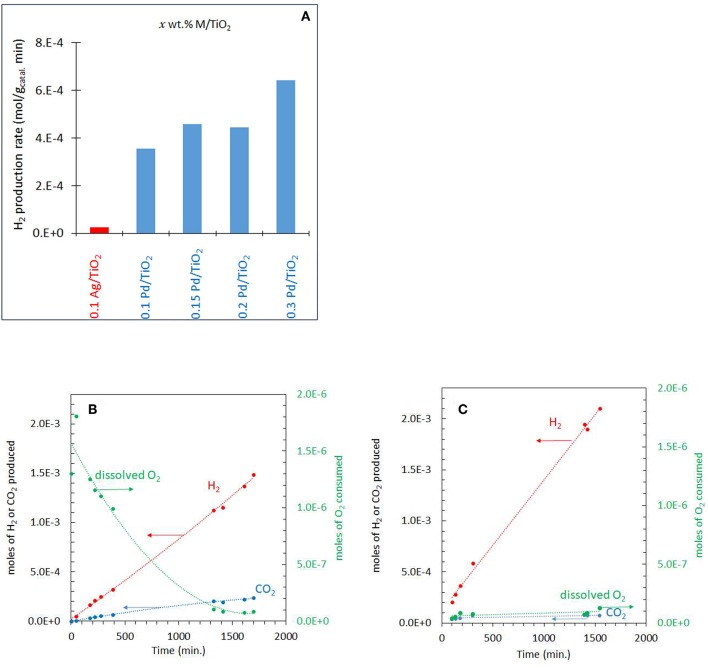
**(A)** Comparison of the performance of the monometallic catalysts for hydrogen production from ethylene glycol at 0.001 atm of O_2_. **(B)** Photoreaction at 0.05 atm *P*_O2_ with 5 vol.% (0.67 M) ethylene glycol and 2 mg of 0.1 wt.% Ag−0.3 wt.% Pd/TiO_2_ (anatase) catalyst. **(C)** Photoreaction at 0.001 atm *P*_O2_ with 5 vol.% (0.67 M) ethylene glycol and 2 mg of 0.1 wt.% Ag−0.3 wt.% Pd/TiO_2_ (anatase) catalyst.

The photo-reforming of ethylene glycol (5 vol.% equivalent to 0.67 M or 0.0134 mole/20 mL which gives a concentration per mass of 6.7 M/g_Catal._) was performed at different partial pressures of O_2_ ranging from 0.001 to 0.20 atm (initial *P*_O2_) over the Ag-Pd/TiO_2_ catalyst. Before presenting them, the activity of the monometallic catalysts was measured and given in Figure 4A. Ag/TiO_2_ has negligible activity while the activity on Pd increased, non-linearly, with Pd%. The role of Ag and Pd together is discussed in the Discussion section. Figure 4B shows the production of H_2_, CO_2_ and consumption of O_2_ during the photoreaction at 0.05 atm *P*_O2_. The rate of H_2_ production (4 x 10^−4^ mol ${\text{g}}_{\text{catal}.}^{-1}$ min^−1^) was very fast when compared to that of CO_2_ (about five times slower, Table 1) which was accompanied by a decrease in O_2_ concentration. Because of the nature of the reaction (slurry), only dissolved O_2_ in the liquid phase would take part in the reaction. Dissolved oxygen was then calculated from the *P*_O2_ using Henry's law during the reaction (Table 1). Due to the low partial pressure of O_2_, reforming of ethylene glycol and oxidation reactions were in competition. The oxidation of ethylene glycol was fast initially due to the presence of O_2_ then the reaction switched to that of reforming. Figure 4C shows the photo-reforming reaction at 0.001 atm. *P*_O2_. The rate of H_2_ was higher when compared to that at 0.05 atm. *P*_O2_ but the rate of CO_2_ changed little. About 95% of the O_2_ was consumed after 1,700 min of reaction.

**Table 1:** H_2_ and CO_2_ production rates and initial concentrations of dissolved O_2_.

***P*_**O2**_ (atm)**	**Initial Dissolved O_**2**_ mol**	**CO_**2**_ mol/g_**catal**_.min**	**H_**2**_ mol/g_**catal**_.min**
0.001	2.60 × 10^−8^	9.60 × 10^−5^	6.40 × 10^−4^
0.02	5.20 × 10^−7^	9.86 × 10^−5^	4.04 × 10^−4^
0.03	7.80 × 10^−7^	9.37 × 10^−5^	3.00 × 10^−4^
0.05	1.30 × 10^−6^	9.45 × 10^−5^	4.22 × 10^−4^
0.10	2.96 × 10^−6^	9.80 × 10^−5^	4.20 × 10^−4^
0.20	5.61 × 10^−6^	9.89 × 10^−5^	3.24 × 10^−4^

*The photoreactions were performed with 5 vol.% ethylene glycol and 2 mg of catalyst. Dissolved oxygen in aqueous solution was calculated by dividing the P_O2_ by Henry's constant 769.23 L atm/mol*.

**Figure 5:**
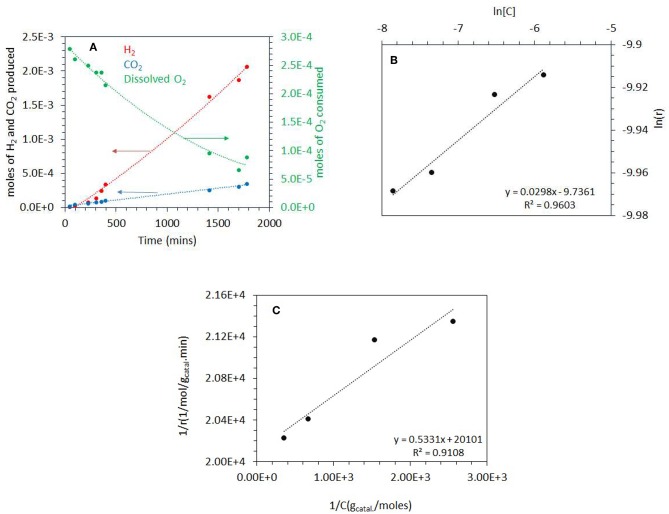
**(A)** Photoreaction performed at 0.2 atm *P*_O2_ with 5 vol.% (0.67 M) ethylene glycol and 2 mg of 0.1 wt.% Ag−0.3 wt.% Pd/TiO_2_ (anatase) catalyst. **(B)** ln [C] vs. ln(r) where C is the concentration of dissolved oxygen and r is the rate of ethylene glycol disappearance. The photoreactions were performed at 0.03–0.20 atm *P*_O2_. The ethylene glycol concentration was 5 vol.% (0.67M) and 2 mg of 0.1 wt.% Ag−0.3 wt.% Pd/TiO_2_ (anatase) catalyst was used for the reaction. **(C)** 1/*r* (g_catal_.min/mol) vs. 1/*C* (g_Catal._/mol), where *C* is the concentration of dissolved oxygen and *r* is the rate of ethylene glycol disappearance. The photoreaction were performed from 0.03 to 0.20 atm *P*_O2_. The ethylene glycol concentration was 5 vol.% (0.67 M) and 2 mg of 0.1 wt.% Ag−0.3 wt.% Pd/TiO_2_ (anatase) catalyst was used for the reaction.

Figure 5A shows the photoreaction at 0.20 *P*_O2_. There was no H_2_ production for the initial 100 min of reaction while CO_2_ was produced with a constant rate. The rate of H_2_ considerably increased after about 500 min concomitant with the decrease in O_2_ concentration and reached about the same level as that reported in Figure 4. These results suggest that at the start of the reaction, the photo-oxidation of ethylene glycol was a dominant reaction and with the decrease in O_2_ concentration, the photo-reforming reaction became predominant. The rates of H_2_ and CO_2_ production from ethylene glycol photoreaction over the range of *P*_O2_ studied are listed in Table 1. The rate of H_2_ production was highest at 0.001 atm *P*_O2_ which is 6 × 10^−4^ mol/g_catal_.min and decreased to 3.2 × 10^−4^mol/g_catal_.min at 0.20 P_O2_. The rate of CO_2_ did not change much by increasing the *P*_O2_ from 0.001 to 0.2 atm. At high *P*_O2_, two further reactions may take place: H_2_-O_2_ recombination to make water on the metal surface and competition between O_2_ and H^+^ to capture excited electrons from the conduction band (O_2_ to ${\text{O}}_{2}^{-.}$ radical and H^+^ to ½ H_2_).

Considering that ethylene glycol is in large excess (0.67 M compared to 2 mg of catalysts and under low light flux ca. 5 mW/cm^2^) the rate of its disappearance by photo-oxidation may be simplified as follows:

(5) $$r=k{\left[O_{2}\right]}^{n}{\left[EG\right]}^{o}{\left[H_{2}O\right]}^{o}$$

(6) $$r=k{\left[O_{2}\right]}^{n}$$

(7) $$lnr=lnk\;+\;nln\left[O_{2}\right]$$

For Equations (5–7), *r* is the rate of disappearance of ethylene glycol, *k* is the rate constant, and *n* is the order of reaction with respect to dissolved O_2_. The rate is zero order with respect to both ethylene glycol and water because they were in large excess. A plot of ln *r* (rate of disappearance of ethylene glycol) and ln [O_2_] (dissolved oxygen concentration in mol/g_Catal._) gives a straight line. We have calculated the rate of disappearance of ethylene glycol from the rate of CO_2_ formation taking into consideration the reaction stoichiometry. This was found to be more accurate than measuring EG concentration directly because it is in excess and therefore its decay was slow (resulting in large error bars). Figure 5B shows the plot of ln *r* as a function of ln [O_2_] for the photo-oxidation reaction. The order of photo-oxidation reaction with respect to dissolved O_2_ concentration was found to be 0.03; in other words almost a negligible dependency (at the tested ethylene glycol concentration).

The Langmuir-Hinshelwood (L-H) kinetic model was further used to study the relation between the rate of photo-oxidation of ethylene glycol to CO_2_ and the binding constant of adsorbed molecular oxygen.

(8) $$r=\frac{kK[C]}{1+K[C]}\;or\;\frac{1}{r}=\frac{1}{k}+\frac{1}{kK[C]}$$

Where *r* represents the rate of photo-oxidation of ethylene glycol, [*C*] the concentration of dissolved O_2_, *k* the rate constant of the reaction and *K* is the adsorption constant or binding constant of O_2_ (Philip Colombo et al., 1995). One usually considers either the initial rate of formation of CO_2_ or the initial rate of disappearance of the organic molecule (Inel and Ökte, 1996). Plotting 1/*r* vs. 1/[*C*] gives a reasonably linear trend Figure 5C (as often the case a deviation occurs at very low concentrations; the last point in the x–axis, 1/[*C*]). The rate constant *k* can then be extracted in addition and found to be = 5 x 10^−5^ mol/g_catal_.min and the binding constant *K* of O_2_ was calculated to be 37,700 g/mol.

Upon photo-excitation, electron transfer from the VB to CB, in the presence of molecular oxygen electron captures occurs resulting in the formation of ${\text{O}}_{2}^{.-}$. This highly oxidizing species, mineralize ethylene glycol to CO_2_ and H_2_O. This is one of the reasons for the decrease of the molecular oxygen concentration with reaction time. This decrease may also be linked to recombination with molecular H_2_ (formed by photo reforming) to make water. The results of this study showed that the oxidation of ethylene glycol was marginal at low *P*_O2_ (Table 1); photo reforming of ethylene glycol is the predominant reaction to form H_2_ and CO_2_. One of the possible reasons for that is the high concentration of ethylene glycol when compared to dissolved oxygen, which results in surface saturation. In other words, electron injections (hole capture) into the valence band would still be the dominant reaction (Fox et al., 1987; Turchi and Ollis, 1990; Mellouki et al., 2003).

### Photoreactions at Low Concentrations of Ethylene Glycol (0.5 vol.%)

The photo-oxidation at higher ethylene glycol concentration showed little dependence on *P*_O2_. In order to study the competition between photo-reforming and photo-oxidation, the reactions were then carried out at low ethylene glycol concentration 0.067 M (this is equivalent to 0.00134 mole/20 mL giving a catalyst concentration of 0.268 M/g_Catal._), the amount of catalyst was increased to 5 mg at *P*_O2_ pressures between of 0.08 to 1 atm. The very small amount of catalyst in the solution does not change light penetration depth and is therefore of no consequences on the light matter interaction.

**Figure 6:**
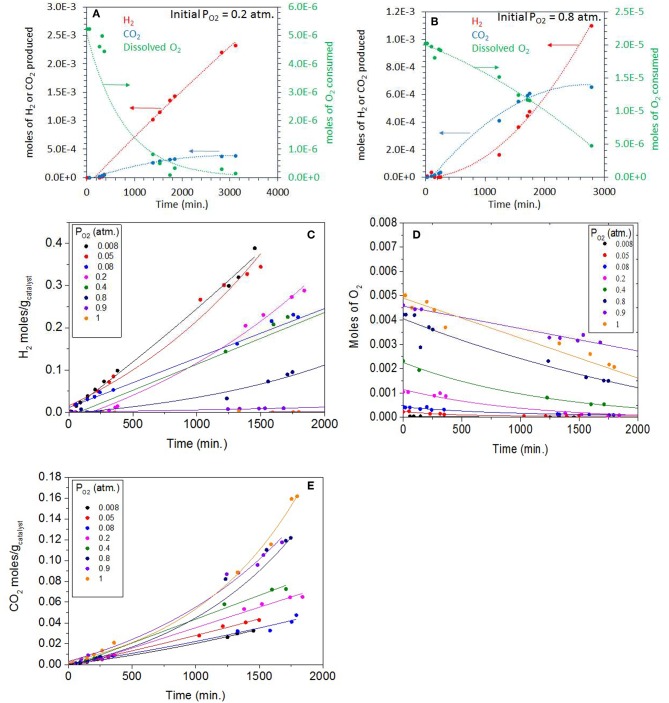
**(A)** Photoreaction performed at 0.20 atm *P*_O2_ with 0.5 vol.% (0.067 M) ethylene glycol and 5 mg of 0.1 wt.% Ag-0.3 wt.% Pd/TiO_2_ (anatase) catalyst. **(B)** Photoreaction performed at 0.8 atm *P*_O2_ with 0.5 vol.% (0.067 M) ethylene glycol and 5 mg of 0.1 wt.% Ag−0.3 wt.% Pd/TiO_2_ (anatase) catalyst. **(C)** H_2_ production during the Photoreaction at 0.008 to 1 atm *P*_O2_ with 0.5 vol.% (0.067 M) ethylene glycol and 5 mg of 0.1 wt.% Ag−0.3 wt.% Pd/TiO_2_ (anatase) catalyst. **(D)** Decrease in O_2_ concentration (gas phase) during the Photoreaction at 0.008–1 *P*_O2_ atm with 0.5 vol.% (0.067 M) ethylene glycol and 5 mg of 0.1 wt.% Ag−0.3 wt.% Pd/TiO_2_ (anatase) catalyst. **(E)** CO_2_ production during the Photoreaction at 0.008–1 *P*_O2_ atm with 0.5 vol.% (0.067M) ethylene glycol and 5 mg of 0.1 wt.% Ag−0.3 wt.% Pd/TiO_2_ (anatase) catalyst.

Figure 6A shows the photoreaction of ethylene glycol at 0.2 atm. *P*_O2_ over the same Ag-Pd-TiO_2_ catalyst. There was no hydrogen production at the start of the reaction with only CO_2_ being formed. H_2_ production started after about 300 min, then the rate considerably increased when dissolved oxygen concentrations dropped exponentially; ca. 95% of which was consumed after 2,000 min of reaction. The rates of CO_2_ slowed down considerably after the full consumption of O_2_, which suggests that CO_2_, initially, was mainly produced by photo oxidation. Based on thermodynamic arguments (Equations 3 and 4) and considering the Brønsted–Evans–Polanyi relation, *E*_a_ = αΔ*H* + β (where E_a_ is the surface reaction activation energy, ΔH is the heat of reaction, α is the BEP proportionality constant, and β is the offset) the photo-oxidation reaction is expected to be faster than the photoreforming reaction.

Figure 6B shows the photoreaction at 0.8 atm *P*_O2_, the rate of CO_2_ was higher in these conditions with no H_2_ being detected. The dissolved oxygen concentration also dropped steadily in favor of CO_2_ production. Due to the high concentration of initially dissolved O_2_, the predominant reaction was the oxidation of ethylene glycol to H_2_O and CO_2_. It is worth noting the inverted relationship between O_2_ consumption and H_2_ production. While at low P_O2_, O_2_ consumption was exponential with a linear H_2_ production with time, at high P_O2_, O_2_ consumption was linear while that of H_2_ production was exponential, with time. This is most likely because of the relation between H_2_ and O_2_ at high P_O2_.

Figure 6C shows H_2_ production during the photoreaction of ethylene glycol as function of time for the whole range of molecular oxygen investigated (from 0.008 to 1 atm. initial *P*_O2)_. As expected the rates of H_2_ production were highest at low *P*_O2_ due to the predominant photo-reforming reaction while at high *P*_O2_ (0.8–1 atm.) the rates were lower because of photo-oxidation. Table 2 shows the rates of H_2_, CO_2_ and the initial concentrations of dissolved oxygen present in the reaction for this series. The slow production of H_2_ at higher *P*_O2_ indicated the competition of O_2_ with H^+^ ions for the capture of conduction band electrons. Figure 6D shows the gradual decrease of dissolved O_2_ during the photoreaction in the 0.008-1 atm initial *P*_O2_. To appreciate the effect one needs to see the ratio of dissolved molecular O_2_ to that of ethylene glycol concentration (Table 2). At low *P*_O2_, the ratio is very high, and the number of moles of oxygen taking part in the photo-oxidation of ethylene glycol was therefore low. At higher *P*_O2_, the low ratio of ethylene glycol to dissolved oxygen is favoring complete oxidation. Figure 6E shows CO_2_ production during the reaction at different *P*_O2_. The rate increased 1.5 and 3.8 times moving from 0.2 atm (partial photo-oxidation) to 1 atm *P*_O2_ (complete photo-oxidation) respectively.

**Table 2:** H_2_ and CO_2_ production rates and initial concentrations of dissolved O_2_.

***P*_**O2**_ (atm)**	**Initial dissolved O_**2**_ mol**	**CO_**2**_ rate mol/g_**catal**_.min**	**H_**2**_ rate mol/g_**catal**_.min**	**Initial moles of EG/Dissolved O_**2**_**
0.008	2.08 × 10^−7^	1.80 × 10^−5^	2.66 × 10^−4^	8607
0.05	1.30 × 10^−6^	2.36 × 10^−5^	1.90 × 10^−4^	1377
0.08	2.35 × 10^−6^	2.55 × 10^−5^	1.22 × 10^−4^	763
0.20	5.24 × 10^−6^	2.84 × 10^−5^	1.70 × 10^−4^	341
0.40	9.37 × 10^−6^	4.75 × 10^−5^	1.31 × 10^−4^	190
0.80	2.03 × 10^−5^	7.37 × 10^−5^	1.32 × 10^−4^	88
0.90	2.33 × 10^−5^	7.00 × 10^−5^	8.00 × 10^−6^	76
1.0	2.55 × 10^−5^	7.20 × 10^−5^	1.00 × 10^−6^	70

*The photoreactions were performed with 0.5 vol.% (0.067 M) ethylene glycol (EG) and 5 mg of catalyst. Dissolved oxygen in aqueous solution was calculated by dividing the P_O2_ by Henry's constant 769.23 L atm/mol*.

**Figure 7:**
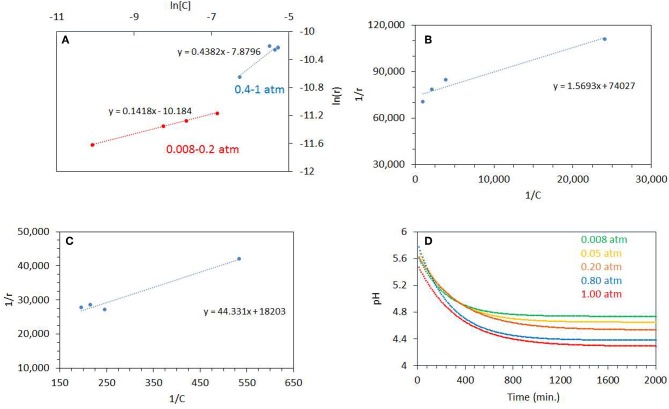
**(A)** ln[*C*] vs. ln(*r*) where *C* is the concentration of dissolved oxygen and *r* is the rate of ethylene glycol disappearance. The photoreactions were performed from 0.08 to 0.20 atm *P*_O2_ and 0.4 to 1 atm *P*_O2_. **(B)** 1/r (g_catal_.min/mol) vs. 1/*C* (g_Catal._/mol), where *C* is the concentration of dissolved oxygen and *r* is the rate of ethylene glycol disappearance. The photoreactions were performed from 0.08 to 0.20 atm *P*_O2_. **(C)** 1/r (g_catal_.min/mol) vs. 1/*C* (g_Catal._/mol), where *C* is the concentration of dissolved oxygen and *r* is the rate of ethylene glycol disappearance. **(D)** Decrease in pH during the Photoreaction at 0.08–1 atm initial *P*_O2_. The line is fitted by exponential decay equation. The ethylene glycol concentration was 0.5 vol.% (0.067 M) and 5 mg of catalyst was used for the reaction.

Figure 7A shows a plot of ln *r* vs. ln [*C*] for the photo-oxidation reactions (where *C* is the concentration of dissolved oxygen and *r* is the rate of ethylene glycol disappearance). The slope gives the order of ethylene glycol disappearance with respect to dissolved oxygen concentration. The order of reaction was 0.14 for the reaction performed at the *P*_O2_ range 0.08–0.20 atm and 0.44 for the 0.4–1 atm range. This shows that there are two distinct regime, in one region CO_2_ production (disappearance of ethylene glycol) is not too affected by *P*_O2_ but at higher *P*_O2_, the order of the reaction approaches ½. Further, Langmuir-Hinshelwood expression was performed by plotting 1/r vs. 1/[C] as shown in Figures 7B,C. The binding constant, *K*_EG_ of O_2_ under illuminations condition was calculated from the slope of the straight line. These were 46,180 g/mol at 0.08–0.20 atm. *P*_O2_ and 410 g/mol for 0.40–1 atm. *P*_O2_. The binding constant is simply the equilibrium of a forward and reverse reaction at given temperature and is not expected to change with the reactant concentration (ΔG = –*RT* ln *K*_EG_):

$$\text{Surface site }+\;{\text{O}}_{2}↔\;\ll \text{Surface site}-{\text{O}}_{2}\;\gg$$

The apparent change in the computed binding constant with the catalyst surface in aqueous environment may be linked to change in the ionic strength of the media, driven by the reaction itself. The change in pH can be estimated by using the concentration of dissolved CO_2_ during the reaction. The concentration of dissolved CO_2_ can be computed from its partial pressure. CO_2_ when dissolved in water forms H_2_CO_3_ (carbonic acid) which is in equilibrium with CO_2_ in water (Equation 9)

(9) $${\text{K}}_{\text{diss}}=\;{\left[{\text{H}}^{+}\right]}^{2}/[{\text{CO}}_{2(\text{aq}.)}]$$

Where K_diss_ is the dissociation constant of H_2_CO_3_ = 4.45 × 10^−7^ at 25°C. Figure 7D shows the pH profiles during the photo- reaction of ethylene glycol. The plots were fitted with an exponential decay function. The fitted equations were then used to model the pH profile with a time interval of 10 min. The pH of the reaction mixture decreased to pH 4.5 after a reaction time of 500 min, for reactions performed at higher *P*_O2_ of 0.8–1 atm., whereas at low *P*_O2_ it decreased to pH 5. The estimated pH (by taking into account dissolved CO_2_) is higher than the actual pH measured after the reaction. This may indicate that the drop in pH was, in addition, due to the formation of other products. The pH of the solution affects the adsorption of organic molecules, reaction rates, extent of adsorption, and the degree of dissociation of organic molecules. TiO_2_ has a zero point charge at 6.5; i.e., it becomes positively charged below 6.5 pH and negatively charged at higher pH affecting the electrostatic interaction between organic molecules and the surface (Friedmann et al., 2010). It has been reported that for the oxidation of ethanol over TiO_2_ the rate of CO_2_ production is low at higher pH. It was also reported that mineralization of alcohols is pH dependent and production of CO_2_ takes place mostly in acidic pH (Chen et al., 1999).

### Liquid Phase Analysis

To further investigate the reaction, we have monitored the liquid-phase intermediates during the photo-reforming of ethylene glycol by GC-MS. For the identification and quantification of liquid intermediates, the reaction was performed at higher initial concentration of ethylene glycol (5 vol.%, 0.67 M) with 50 mg of catalyst at 0.0025 atm. *P*_O2_. The increased amount of catalyst here is to ensure quantifications of the reaction products with minimal experimental errors.

**Figure 8:**
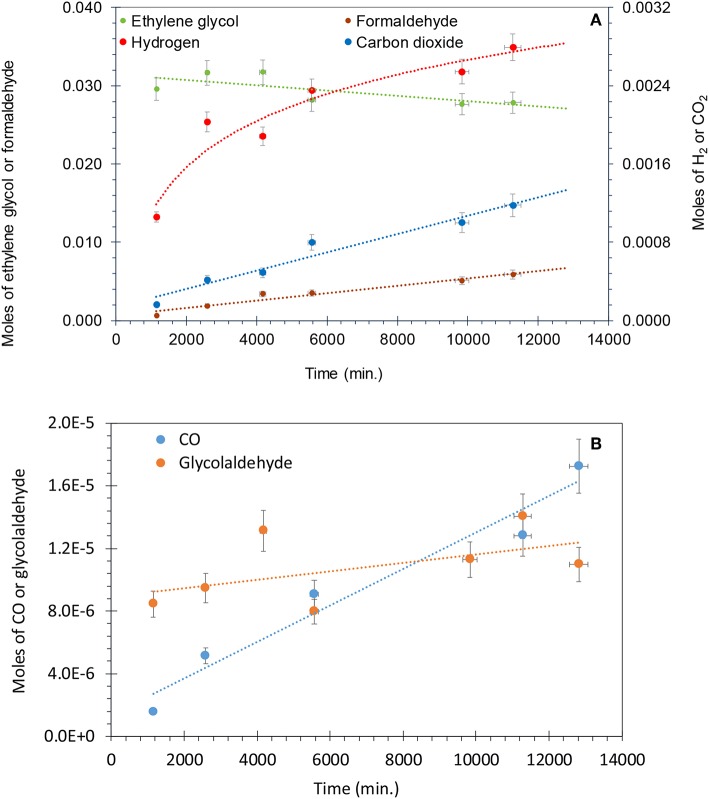
**(A)** Photo-reforming of ethylene glycol 5 vol.% (0.67 M) with 50 mg of 0.1 wt.% Ag−0.3 wt.% Pd/TiO_2_ (anatase) catalyst. Formaldehyde and ethylene glycol concentration are from the liquid phase, while H_2_ and CO_2_ from the gas phase. **(B)** Production of CO and glycol aldehyde during the photo-reforming of ethylene glycol 5 vol.% (0.67 M) with 50 mg of 0.1 wt.% Ag−0.3 wt.% Pd/TiO_2_ (anatase) catalyst.

Figure 8A shows that for the photo-reforming of 5 vol.% ethylene glycol, H_2_ and CO_2_ were the main products in the gas phase while CO was also detected in trace amounts. The production of H_2_ slows down while the CO_2_ rate was not much affected_._ The intermediates detected in the liquid phase were formaldehyde and glycol aldehyde, similar to other studies (Nishimoto et al., 1985). Formaldehyde was the main intermediate and its concentration increased with reaction time, reaching 6 mmol after 11,000 min. The total moles of formaldehyde in the liquid phase were higher than that of H_2_ (4 mmol) and CO_2_ (1.2 mmol) in the gas phase after the completion of the reaction. This indicates that formaldehyde is the main intermediate product in the photo-reforming reaction.

Figure 8B presents the production of CO and glycol aldehyde; CO was detected in very low concentrations and it increased gradually with reaction time. Glycol aldehyde concentration fluctuated during the reaction, most likely because it quickly converted to other products without accumulating in the liquid phase and it was present in very low concentrations; ca. 10 μmol in the liquid phase after the completion of the reaction. It has been reported that formyl groups containing oxygenates such as formaldehyde and glycol aldehyde produces high concentration of CO (Shabaker and Dumesic, 2004). Returning to Figure 8A, a slight oscillation of the EG concentration is noticed in the beginning of the reaction, most likely because a desorption of EG from the walls of the reactor initially. Then EG concentration decreased with time. Focusing on the last three points we can extract the mass balance of the reaction taking into consideration the observed reaction products and the amount of EG that has disappeared. This is presented in Table 3. As can be seen in the last column the deviation from a complete mass balance decreases with reaction time. At the end of the reaction it appears that the sum of weight of formaldehyde + CO_2_ + H_2_ + GA + CO is equal to the amount of EG that has disappeared. The fact that the reaction takes long time to reach this point indicates that between GA and formaldehyde other intermediate products are formed that were not detected during the experiment.

**Table 3:** Mass balance extracted from the last points of Figure 8A.

**Time**	**Mole**	**Mole**	***g***	**Mole**	***g***	**Mole**	***g***	**Mole**	***g***	**Mole**	***g***	**Mole**	***g***	***g***	
**Minutes**	**EG**	**^a^EG consumed**	**EG consumed**	**H_**2**_**	**H_**2**_**	**CO_**2**_**	**CO_**2**_**	**HCHO**	**HCHO**	**^b^GA**	**GA**	**CO**	**CO**	**Total products**	**^c^% difference**
5,568	0.02819	0.00348	0.21560	0.00235	0.00470	0.00080	0.03512	0.00353	0.10583	0.00001	0.00048	0.00001	0.00025	0.14638	32.1%
9,838	0.02766	0.00402	0.24893	0.00254	0.00509	0.00100	0.04412	0.00511	0.15329	0.00001	0.00068			0.20318	18.4%
11,278	0.02784	0.00383	0.23764	0.00279	0.00559	0.00118	0.05183	0.00590	0.17706	0.00001	0.00084	0.00001	0.00036	0.23568	0.8%

a *EG, ethylene glycol*.

b *GA, glycolaldehyde*.

c *%difference = (consumed EG – total products)/consumed EG) × 100*.

Equations (10–15) show the possible reaction pathway of ethylene glycol reforming.

(10) $${\text{C}}_{2}{\text{H}}_{6}{\text{O}}_{2}\;+\;2\text{h }+\;2\text{e}\to {\text{C}}_{2}{\text{H}}_{4}{\text{O}}_{2}\;+\;{\text{H}}_{2}$$

(11) $${\text{C}}_{2}{\text{H}}_{4}{\text{O}}_{2}\to 2\text{HCHO}$$

(12) $$2\text{HCHO}\to 2\text{CO }+\;2{\text{H}}_{2}$$

(13) $$2\text{CO }+\;2{\text{H}}_{2}\text{O}\to 2{\text{CO}}_{2}\;+\;2{\text{H}}_{2}$$

The sum of Equations (10–13) gives Equation (3)

(14) $$\text{HCHO }+\;{\text{H}}_{2}\text{O }+\;2\text{h}\to \text{HCOOH }+\;2{\text{H}}^{+}$$

(15) $$\text{HCOOH}\to \text{CO }+\;{\text{H}}_{2}\text{O}$$

Ethylene glycol directly injects electrons into the valence band of TiO_2_ and dehydrogenates to glycol aldehyde, Equation (10). Glycol aldehyde undergoes C-C bond scission to give 2 moles of formaldehyde, Equation (11). Then, Formaldehyde decomposed to CO and H_2_, equation 12. CO formed in reaction 12 is consumed by WGSR to give H_2_ and CO_2_, Equation (13). Alternatively formaldehyde may also photo-reform to give formic acid and H_2_, dehydration of formic acid results in CO and H_2_O (Puga, 2016; Equations 14, 15). Haruta and collaborators have shown that WGSR can take place on Au-TiO_2_ at 100°C with a similar activity to that of traditional Cu/ZnO catalysts (Sakurai et al., 1997). There is not much literature available on photocatalytic water gas shift reaction with scarce reaction mechanism (Sakurai et al., 1997; Millard and Bowker, 2002; Sandoval et al., 2007; Sastre et al., 2013). Berto et al. studied the photo-reforming of ethylene glycol, glycol aldehyde, formaldehyde and formic acid over TiO_2_ and observed that the largest amounts of CO were produced by formic acid (Berto et al., 2016). For the photo-reforming of glycerol over 1 wt.%Pt-TiO_2_, Panayiotopoulos et.al proposed the breaking of C-C bond of glycol aldehyde to yield methanol and CO followed by the oxidation of methanol to formaldehyde and H_2_ (Panagiotopoulou et al., 2013; Lu et al., 2014). In this study, neither methanol nor acetaldehyde were observed in the liquid and gas phase, which means that glycol aldehyde dissociated mainly by C-C bond scission to give formaldehyde that further oxidized to formic acid or formates. However, formic acid was not detected in this study either, which may be due to the strong dissociative adsorption of formic acid to formates.

At low concentrations of organics such as glycerol, photo-oxidation mainly takes place by H abstraction while at high concentrations direct electron injection into the valence band is the main reaction (Minero et al., 2012). The rate constants for the reaction of OH^**·**^ radicals with diols are higher than that of the mono alcohols, which shows the effect of second OH group. It has been reported that the abstraction of a hydrogen atom from an α-carbon atom by a hydroxyl group is enhanced by the presence of second hydroxyl group at the β-carbon position. The main products from the OH^**·**^ radical initiated oxidation of diols are the corresponding hydroxyl ketones. The hydrogen atom abstraction takes place by CH_2_OH or -CHOH groups in these molecules followed by reaction of corresponding α hydroxyl alkyl radicals with O_2_ to give the corresponding α-hydroxy carbonyl compounds (Bethel et al., 2001; Yujing and Mellouki, 2001).

In this work, the conversion of ethylene glycol after photo-reforming for 11,000 min was 15.7%. The selectivity of H_2_, CO_2_, formaldehyde, glycol aldehyde, and CO were 19, 13.4, 67, 0.4, and 0.3%, respectively. The low H_2_ selectivity could be due to the consumption of H_2_ through hydrogenation of some of these intermediates. High concentrations of formaldehyde in the liquid phase also indicated the possibility of strong CO chemisorption on Pd sites that can limit further conversion of formaldehyde. Strong adsorption of formic acid compared to formaldehyde can also limits the conversion of formaldehyde. It was reported that under illumination condition the adsorption constant of formaldehyde was 2 orders of magnitudes lower than that of formic acid (Berto et al., 2016). Shabaker et al. showed that aqueous phase reforming of methanol and ethylene glycol gives similar hydrogen production rates over Pt/Al_2_O_3_. They proposed that the cleavage of the C–C bond in ethylene glycol is not a rate-limiting step and ethylene glycol complete reforming may be limited by C–H, O–H bond cleavage, formation of dehydrogenated intermediates and removal of adsorbed CO from the metal surface by WGSR (Shabaker and Dumesic, 2004). Similar conclusions were deduced by Berto et al. for photo-reforming of ethylene glycol and glycol aldehyde over Rh-TiO_2_. Glycol aldehyde gives higher rates of H_2_ compared to ethylene glycol; this is because glycol aldehyde conversion reaction is initiated by the C-C bond cleavage whereas in ethylene glycol, the OH group is oxidized first. These results suggested that C-C bond cleavage is not rate limiting step for the photo-reforming reaction (Berto et al., 2016).

## Discussion

The photo-reforming of ethylene glycol gives H_2_ and CO_2_ along with products in the liquid phase. The ratio of H_2_ to gas phase CO_2_ was higher than the stoichiometric ratio of 2.5, which means that part of CO_2_ remained dissolved and/or there is an accumulation of secondary products. CO was not observed at higher *P*_O2_ reactions due to oxidation, and was only observed in photo-reforming conditions. Due to the higher *P*_O2_, the backward reaction of H_2_ and O_2_ recombination became prominent. The Langmuir kinetics for ethylene glycol consumption showed linear trends and its disappearance (calculated from CO_2_ production) increased with the increase of dissolved O_2_ concentration.

Figures 5A, 6A show photoreaction performed in air at 0.2 *P*_O2_ at two different ethylene glycol concentrations, 0.67 and 0.067 M, where no hydrogen production was seen for 100 min and 400 min, respectively. The positive effect of increasing the ethylene glycol concentration over the H_2_ production suggests that even at higher *P*_O2_, H_2_ production reaction was not retarded. This indicated that the competition between photo reforming and photo-oxidation depends on initial concentrations of oxygen and ethylene glycol. Increasing the concentration of ethylene glycol does not linearly increase the reaction rate probably because the rate is limited by the number of incident photons, in the present study.

It has been reported that photo-oxidation in the presence of O_2_ proceeds by the formation of O_2_ superoxide which also yields other peroxide species such as H_2_O_2_ and HO_2_^**·**^ radicals (Linsebigler et al., 1995; Wang et al., 2002). Daskalaki et al. identified the production of peroxides during the photo-reforming of glycerol over a Pt-TiO_2_ catalyst where it was suggested that the primary oxidizing species is the OH^**·**^ radical (Daskalaki et al., 2011).

There are several factors that might cause the gradual decrease of H_2_ production rate in photo-reforming of ethylene glycol, such as accumulation of reaction intermediates in the liquid phase, buildup of surface bound species (formates and carbonates) and the strong chemisorption of CO. The higher selectivity to formaldehyde during photo-reforming of ethylene glycol indicates that the reaction was not completed even after 11,000 min. Therefore, the accumulation of intermediates resulted in incomplete photo-reforming reaction and decreased the overall selectivity to H_2_.

The main purpose of reforming of glycols and alcohols is to get H_2_. The product distribution of the reforming reaction also depends upon the type of metal on the support. In this study 0.1 wt.% Ag−0.3 wt.% Pd was used, Pd has a relatively higher C-C bond scission activity when compared to Ag and is also active for formic acid decomposition (Bahruji et al., 2013). CO has higher binding constant over Pd surface then Ag and due to this strong chemisorption, WGSR over Pd-TiO_2_ in ambient condition is relatively slow (Kandoi et al., 2004; Huber et al., 2006). There are also some parallel reactions such as methanation and reverse WGSR that decrease the selectivity to H_2_ (Luo et al., 2008). To improve the H_2_ selectivity the catalyst must achieve less selectivity for alkanes and should be active for C-C bond cleavage in addition to being active for the WGSR (He et al., 2005). Reforming reaction kinetics by others indicated that there is not only inhibition reaction due to the presence of CO but H_2_ also blocks the active sites to retard the reaction with negative order (Huber et al., 2006).

Alloying noble metals such as Pt with Co and Ni can shifts the d-band center of Pt by 0.5–0.7 eV which also affects the binding energy of the molecules over the alloyed metal (Christoffersen et al., 2001). It has been observed that by alloying Pt with Ni and Co, there is a decrease in the adsorption energy for H_2_ and CO (Greeley and Mavrikakis, 2004). Alloying Pd with Ag can also decrease the adsorption energy of CO even at low surface coverage (Christmann and Ertl, 1972). The presence of Ag and Pd as an alloy in our study may have assisted the WGSR due to the low binding energy of CO over the “most likely” Ag-Pd alloy catalyst. Similar observations were made by others where enhanced photo-reforming of glycerol was observed over bimetallic Au-Pt/TiO_2_ catalyst under UV light and the rates of H_2_ were higher than those of the mono metallic catalysts, the enhancement was attributed to the nature of the Pt-Au alloy which facilitates desorption of H_2_ (Tanaka et al., 2013).

## Conclusions

While molecular hydrogen can be produced by the photocatalytic reforming of ethylene glycol over Ag-Pd/TiO_2_ in high yields, the presence of molecular oxygen changes the reaction network. At low concentrations of ethylene glycol, the photo-oxidation to CO_2_ and water dominated particularly at high partial pressures of O_2_. At high concentrations of ethylene glycol, hydrogen production, via photoreforming, was far less sensitive to the partial pressure of O_2_. The order of the photoreaction of 0.5 vol.% ethylene glycol with respect to dissolved oxygen concentrations was 0.14 and 0.44 at low (<0.2 atm.) and high (between [0.4–1 atm.]) *P*_O2_, respectively. Liquid phase analysis of the photo-reforming reaction showed formaldehyde to be the main reaction intermediate along with glycol aldehyde. Glycol aldehyde is proposed to give formaldehyde and CO by C-C bond cleavage. CO then reacts with water to give CO_2_ and H_2_ in a photo-stimulated water gas shift reaction. The presence of high concentrations of formaldehyde (higher than those of hydrogen and CO_2_) indicates that the photo reforming of ethylene glycol was incomplete.

## Data Availability Statement

The datasets generated for this study are available on request to the corresponding author.

## Author Contributions

AW conducted the experimental work, wrote the first draft. HI, principle investigator, conceived the work, further analyzed the data and worked on subsequent versions of the manuscript. MN conducted Transmission Electron Microscopy (TEM) and High Resolution TEM.

### Conflict of Interest

The authors declare that the research was conducted in the absence of any commercial or financial relationships that could be construed as a potential conflict of interest.
